# Birth length is the strongest predictor of linear growth status and stunting in the first 2 years of life after a preconception maternal nutrition intervention: the children of the Women First trial

**DOI:** 10.1093/ajcn/nqac051

**Published:** 2022-06-10

**Authors:** Nancy F Krebs, K Michael Hambidge, Jamie L Westcott, Ana L Garcés, Lester Figueroa, Antoinette K Tshefu, Adrien L Lokangaka, Shivaprasad S Goudar, Sangappa M Dhaded, Sarah Saleem, Sumera Aziz Ali, Melissa S Bauserman, Richard J Derman, Robert L Goldenberg, Abhik Das, Dhuly Chowdhury, Women First Preconception Maternal Nutrition Study Group, Vanessa R Thorsten, Amaanti Sridhar, Elizabeth McClure, Veena Herekar, S Yogeshkumar, Sunil S Vernekar, Manjunath Somannavar, Carl L Bose, Marion Koso-Thomas

**Affiliations:** Department of Pediatrics, Section of Nutrition, University of Colorado School of Medicine, Denver, CO, USA; Department of Pediatrics, Section of Nutrition, University of Colorado School of Medicine, Denver, CO, USA; Department of Pediatrics, Section of Nutrition, University of Colorado School of Medicine, Denver, CO, USA; Unidad de Salud Materno Infantil, Instituto de Nutrición de Centroamérica y Panamá (INCAP), Calzada Roosevelt, Guatemala City, Guatemala; Unidad de Salud Materno Infantil, Instituto de Nutrición de Centroamérica y Panamá (INCAP), Calzada Roosevelt, Guatemala City, Guatemala; Kinshasa School of Public Health, Hôpital Général de Kinshasa, Kinshasa, Democratic Republic of the Congo; Kinshasa School of Public Health, Hôpital Général de Kinshasa, Kinshasa, Democratic Republic of the Congo; KLE Academy of Higher Education and Research, Jawaharlal Nehru Medical College, Belagavi, India; KLE Academy of Higher Education and Research, Jawaharlal Nehru Medical College, Belagavi, India; Department of Community Health Sciences, Aga Khan University, Karachi, Pakistan; Department of Community Health Sciences, Aga Khan University, Karachi, Pakistan; Department of Pediatrics Neonatal-Perinatal Medicine, University of North Carolina, Chapel Hill, NC, USA; Department of OBGYN, Thomas Jefferson University, Philadelphia, PA, USA; Department of OBGYN, Columbia University Medical Center, New York, NY, USA; RTI International, Durham, NC, USA; RTI International, Durham, NC, USA

**Keywords:** stunting, breastfeeding, growth, infant growth, maternal height, preconception, birth length

## Abstract

**Background:**

The multicountry Women First trial demonstrated that nutritional supplementation initiated prior to conception (arm 1) or early pregnancy (arm 2) and continued until delivery resulted in significantly greater length at birth and 6 mo compared with infants in the control arm (arm 3).

**Objectives:**

We evaluated intervention effects on infants’ longitudinal growth trajectory from birth through 24 mo and identified predictors of length status and stunting at 24 mo.

**Methods:**

Infants’ anthropometry was obtained at 6, 12, 18, and 24 mo after the Women First trial (registered at clinicaltrials.gov as NCT01883193), which was conducted in low-resource settings: Democratic Republic of Congo, Guatemala, India, and Pakistan. Longitudinal models evaluated intervention effects on infants’ growth trajectory from birth to 24 mo, with additional modeling used to identify adjusted predictors for growth trajectories and outcomes at 24 mo.

**Results:**

Data for 2337 (95% of original live births) infants were evaluated. At 24 mo, stunting rates were 62.8%, 64.8%, and 66.3% for arms 1, 2, and 3, respectively (NS). For the length-for-age *z*-score (LAZ) trajectory, treatment arm was a significant predictor, with adjusted mean differences of 0.19 SD (95% CI: 0.08, 0.30; *P* < 0.001) and 0.17 SD (95% CI: 0.07, 0.27; *P* < 0.001) for arms 1 and 2, respectively. The strongest predictors of LAZ at 24 mo were birth LAZ <–2 and <–1 to ≥–2, with adjusted mean differences of –0.76 SD (95% CI: –0.93, –0.58; *P* < 0.001) and –0.47 SD (95% CI: –0.56, –0.38; *P* < 0.001), respectively. For infants with ultrasound-determined gestational age (*n* = 1329), the strongest predictors of stunting were birth LAZ <–2 and <–1 to ≥– 2: adjusted relative risk of 1.62 (95% CI: 1.39, 1.88; *P* < 0.001) and 1.46 (95% CI: 1.31, 1.62; *P* < 0.001), respectively.

**Conclusions:**

Substantial improvements in postnatal growth are likely to depend on improved intrauterine growth, especially during early pregnancy.

See corresponding editorial on page 1.

## Introduction

The multicountry Women First (WF) trial demonstrated that comprehensive nutritional supplementation initiated at least 3 mo prior to conception or at the end of the first trimester resulted in significantly longer birth length (the primary outcome) and greater birth weight. Rates of stunting at birth, low birth weight, and small for gestational age were also significantly reduced in the intervention arms compared with a nonintervention control group (1). Despite the nutrition intervention being stopped at delivery, the favorable growth effects on linear and ponderal growth were sustained through 6 mo, and stunting rates at 6 mo were lower for the preconception and early pregnancy intervention arms (2). The plausibility of these observations is supported by recognition that the physiologic processes during early gestation are particularly critical for linear growth, have long-term programming effects, and are exquisitely sensitive to maternal nutritional status and well-being (3, 4).

The potential for persistence of such preconception and prenatal effects on linear growth faltering during the complementary feeding period, that is, over the second half of the 1000 d, is less clear. Although declining, the overall prevalence of stunting in children <5 y of age remains in the 20–25% range, with rates varying substantially among regions of the globe (5, 6). A recent “roadmap” to reduce stunting identified multiple health and nonhealth factors that have been associated with improvements in postnatal linear growth and reductions in stunting rates. These factors prominently included several maternal factors, including improved nutrition (7).

Thus, the goal of the analyses in this report was to determine whether the benefits of improved maternal nutritional status prior to conception and during early gestation on fetal and early postnatal growth were sustained at 2 y. The objectives of these analyses were 3-fold: *1*) to examine postnatal growth and whether the benefits of the intervention evident at birth and 6 mo were detectable through the first 24 mo after birth, *2*) to identify predictors of the longitudinal growth trajectory from birth through 24 mo of age, and *3*) to identify, in a cross-sectional analysis, the major predictors of linear growth status at 24 mo for infants of all participating women and separately for the subgroup of infants for whom gestational age determinations were available.

## Methods

### Study design and settings

This analysis included prospectively obtained anthropometry of those live-born infants of the women in the WF trial (registered at clinicaltrials.gov as NCT01883193) who had birth measurements and who consented to the follow-up growth monitoring study at 6 mo and continued through 24 mo postnatal age. Growth from birth to 6 mo was an a priori secondary outcome of the primary trial, and results have been reported elsewhere (1, 2). In this report, we examined infants’ trajectory of linear and ponderal growth over the entire interval from birth through 24 mo according to treatment arm of the mother, and we determined predictors of linear growth status at 24 mo.

The original trial was a multisite, individually randomized, clinical trial in which nonpregnant women were randomly assigned to 1 of 3 arms: initiation of a daily small quantity lipid-based nutrient supplement (SQ-LNS) at the time of randomization with continuation for at least 3 mo prior to conception through to delivery (arm 1), initiation of the same supplement late in the first trimester of pregnancy and continued through to delivery (arm 2), or receipt of no trial supplement (arm 3). Women in arm 1 and arm 2 (once having started the primary supplement) who were underweight or had inadequate gestational weight gain were provided an extra protein-energy supplement until delivery. More than 85% of the participating women in 3 of the sites received the protein-energy supplement; <10% of the Guatemalan women qualified for the second supplement. The trial was conducted in low-resource, rural, and small city settings in 4 countries: Democratic Republic of the Congo (DRC), Guatemala, India, and Pakistan (1, 8).

### Participants

Consent was obtained from the WF participants to be contacted for ongoing measurements and data collection for their live-born infants. Birth anthropometric measurements, including length, weight, and head circumference, were obtained by study assessment teams by at least 7 d of postnatal life, and 98.2% were obtained within 48 h of birth. Anthropometric measurements were obtained at 6, 12, 18, and 24 mo of age between February 2015 and March 2019. Because of interest in gestational age-dependent covariates [e.g., small for gestational age (SGA), prematurity], we examined potential predictors from the subgroup of infants for whom gestational age was determined by first-trimester ultrasounds; ultrasound capacity was not available in the DRC.

### Randomization

The central data coordinating center (DCC; RTI International) created the randomization scheme for the original trial, generating the allocation sequence separately for each research site. To ensure geographic balance, a permuted block design stratified by geographical clusters was used to generate the randomization sequence for assigning individual participants to a trial arm. The allocation ratio was 1:1:1 within blocks, which randomly varied between sizes of 3, 6, or 9. At each site, once the responsible home visitor research assistant (HVRA) identified an eligible participant, the HVRA then received the randomization assignment from their site-specific data manager, who generated the randomization assignment from the centralized computerized data management system maintained by the DCC.

### Anthropometry

Length, weight, and head circumference measurements were obtained by assessment teams who were not directly involved in administration of the study intervention or any of the home visits throughout the trial. The assessors were trained and certified according to standardized procedures and were recertified every 3 mo. The equipment included electronic balances accurate to 10 g (seca 334; seca North America); nonstretch, plasticized measuring tapes (seca 201; seca North America); and length boards accurate to 1 mm (neonatal and pediatric stadiometers; Ellard Instrumentation, Ltd). The *z*-scores, which accounted for sex and age at the time of measurement, were calculated for length-for-age *z*-score (LAZ), weight-for-age *z-*score (WAZ), weight-for-length *z*-score (WLZ), and head circumference-for-age *z*-score from the WHO Child Growth Standards (9). For women who had first-trimester ultrasounds performed, the gestational age-specific birth anthropometric *z*-scores were also determined based on INTERGROWTH-21st fetal growth charts (10).

### Ethics

The project was approved by the Colorado Multiple Institutional Review Board, University of Colorado; the local and/or national ethics committees for each of the 4 sites (registered with the US Office of Human Research Protection and with federal-wide assurance in place); and the DCC (RTI International). Written informed consent was obtained from all participants. The study protocol is available online: https://www.ncbi.nlm.nih.gov/pmc/articles/PMC4000057/. Throughout the intervention phase of the trial, a data monitoring committee designated by the *Eunice Kennedy Shriver* National Institute of Child Health and Human Development monitored the safety of the trial. Adverse events that were monitored included pregnancy outcomes, adverse neonatal events, hospitalizations, and allergic reactions (1, 8).

### Statistics

#### Overall approach and model selection

For all the adjusted models, collinearity of independent variables was examined using Spearman and Pearson correlation coefficients for discrete and continuous variables, respectively. In addition to reviewing the correlations, *R*^2^ and comparisons of log-likelihoods were also considered for final model selection. In addition, interactions between arm and site, sex and arm, and arm and parity were examined.

Some of the continuous predictors (height and BMI) were categorized based on the cutoff values identified by classification and the regression tree algorithm. Paternal variables were missing for ∼15.5% infants. A separate category for the missing values was created to include these records in all the final models. Paternal height was categorized as a 3-group variable: ≤160 cm, >160 cm, and missing. Similarly, paternal BMI (in kg/m^2^) was categorized as ≤24, >24, and missing.

Two-sided *P* values were reported throughout, with *P* < 0.05 considered statistically significant. Statistical analyses were conducted using SAS 9.4 statistical software (SAS Institute).

#### Maternal treatment effects on postnatal longitudinal growth

After excluding biologically implausible anthropometric measurements (length and head circumference) according to WHO guidelines, the longitudinal analysis included all live-born infants with birth length measurements obtained by 7 d of age who were alive at 6 mo and whose parents had provided consent for follow-up to 2 y. We constructed longitudinal models using generalized estimating equations (GEEs) to examine whether the trajectory of growth for study infants from birth through 24 mo age differed by treatment arms. Baseline maternal characteristics that differed across the 3 treatment arms, or between infants included or excluded from the analysis due to missing data, were considered for inclusion as covariates in these models: socioeconomic status (SES), maternal education, parity, BMI, height, and stunting. Model selection approaches described above were used to develop the final model. Continuous (binary) outcomes assumed a normal (binomial) distribution with identity (log) link and an autoregressive correlation structure with robust sandwich estimator (empirical estimates) to account for infant repeated measures over time. We examined treatment heterogeneity by country using interaction terms for arm and country. Interactions were also examined to assess the stability of treatment effects over time for each model.

#### Predictors of longitudinal growth trajectory

A second series of longitudinal GEE models were fit on the same data to identify the predictors of the longitudinal growth trajectory from birth through 24 mo, adjusting for treatment arm. These models also adjusted for maternal and paternal BMI and height. In addition, interactions between arm and site, arm and visit, sex and arm, and arm and parity were examined and included in the final model, as appropriate.

#### Predictors of linear growth status at 24 mo

We used cross-sectional analyses, linear (robust Poisson) regression for continuous (binary) outcomes, to identify the major predictors of linear growth status at 24 mo. First, for the all-site analysis, the following potential predictors were considered: SES; maternal education, parity, stunting, height, and BMI; paternal height and BMI; and birth measurements of length, LAZ, and stunting, categorized as none (LAZ ≥–1), mild (LAZ <–1 to ≥–2), or moderate (LAZ <–2), and weight, WAZ, WAZ <–2, weight-for-length, WLZ, WLZ <–2, sex, and low birth weight (LBW) using WHO growth standards. After model selection, the final models included arm; site; cluster; interaction between site and cluster; SES; maternal education, parity, BMI (≤22 and >22), and height (≤150 cm, >150 cm); and paternal BMI (≤22, >22, and missing) and height (≤160 cm, >160 cm, and missing).

Separate analyses were conducted for the set of data from India, Pakistan, and Guatemala with available gestational age determined by first-trimester ultrasound. The predictors considered were SES; maternal education, parity, stunting, height, and BMI; paternal height and BMI; and birth measurement *z*-scores derived from INTERGROWTH-21st (IG) fetal growth charts (10) of length; LAZ_IG_; stunting categorized as categorized as none (LAZ_IG_ ≥–1), mild (LAZ_IG_ <–1 to ≥– 2), or moderate (LAZ_IG_ <–2); weight; WAZ_IG_; WAZ_IG_ <–2; weight-for-length; WLZ_IG_; WLZ_IG_ <–2; SGA; preterm birth (<37 wk of gestation); and sex. All these predictors were considered in the adjusted models for each outcome variable. Upon model selection, the final models included arm; site; cluster; interaction between site and cluster; SES; maternal education, parity, BMI (≤22 and >22), and height (≤150 cm, >150 cm); paternal BMI (≤22, >22, and missing) and height (≤160 cm, >160 cm, and missing); infant sex; birth stunting categories; preterm birth; and SGA (not LBW).

## Results

This analysis is based on infants born to 2324 women who participated in the primary WF trial and consented to the follow-up study, for which enrollment and randomization occurred between December 2013 to October 2014 (**Supplemental Figure 1**). Baseline characteristics for the mothers and fathers of the infants and young children of the present analyses are presented in **Table 1**. No differences among arms were evident except that arm 1 had a higher percentage of women with no education and a higher percentage of nulliparous women. The total number of infants considered for the combined site analysis was 2337 (95% of original live births), evenly distributed across arms and sex, with 755, 808, and 774 in arms 1, 2, and 3, respectively (Supplemental Figure 1), and with 1162 males and 1175 females. For the subgroup of infants with gestational age determinations, 1329 infants (Guatemala, 439; India, 487; Pakistan, 403) were included; sample sizes according to treatment arm were 438, 478, and 413 for arms 1, 2, and 3, respectively.

**TABLE 1:** Baseline characteristics among women who had a live birth in the 24-mo longitudinal analysis subset, all sites combined and by treatment arm^1^

Variable	Total	Arm 1	Arm 2	Arm 3	*P* value^2^
Women who had a live birth in the 24-mo longitudinal analysis population, *n*^3^	2324	748	806	770	
Maternal age, *n*	2324	748	806	770	0.398
<20 y	481 (20.7)	146 (19.5)	179 (22.2)	156 (20.3)	
20+ y	1843 (79.3)	602 (80.5)	627 (77.8)	614 (79.7)	
Maternal education, *n*	2324	748	806	770	0.016
No formal schooling	746 (32.1)	265 (35.4)	240 (29.8)	241 (31.3)	
Primary	872 (37.5)	247 (33.0)	313 (38.8)	312 (40.5)	
Secondary +	706 (30.4)	236 (31.6)	253 (31.4)	217 (28.2)	
Maternal BMI, *n*	2323	748	805	770	
BMI, kg/m ^2^, Mean ± SD	21.4 ± 4.0	21.4 ± 4.0	21.4 ± 4.1	21.4 ± 3.9	0.997
BMI ≤22.0	1509 (65.0)	493 (65.9)	524 (65.1)	492 (63.9)	0.710
BMI <18.5	546 (23.5)	177 (23.7)	194 (24.1)	175 (22.7)	0.808
Maternal height, *n*	2323	748	805	770	
Height, cm, Mean ± SD	151.4 ± 6.9	151.7 ± 6.5	151.3 ± 7.0	151.3 ± 7.0	0.506
Height ≤150.0 cm^4^	995 (42.8)	299 (40.0)	364 (45.2)	332 (43.1)	0.111
Parity, *n*	2324	748	806	770	0.014
0 (nulliparous)	474 (20.4)	179 (23.9)	153 (19.0)	142 (18.4)	
≥1	1850 (79.6)	569 (76.1)	653 (81.0)	628 (81.6)	
Paternal height, *n*	1954	643	666	645	
Height, cm, Mean ± SD	163.5 ± 7.7	163.6 ± 7.9	163.4 ± 7.5	163.5 ± 7.6	0.935
Paternal BMI, *n*	1953	643	666	644	
BMI, kg/m ^2^, Mean ± SD	22.0 ± 3.7	22.1 ± 3.9	21.9 ± 3.7	21.9 ± 3.6	0.605
Height ≤160.0 cm	650 (33.3)	209 (32.5)	222 (33.3)	219 (34.0)	0.858
Tally of indicators of higher SES^5^,*n*	2324	748	806	770	
Median (P25–P75)	3.0 (1.0, 4.0)	3.0 (1.0, 4.0)	3.0 (1.0, 4.0)	3.0 (1.0, 4.0)	
Low (0–2 present)	985 (42.4)	331 (44.3)	332 (41.2)	322 (41.8)	0.441
High (3–6 present)	1339 (57.6)	417 (55.7)	474 (58.8)	448 (58.2)	

1 Values are presented as number (%) unless otherwise indicated. Arm 1 maternal participants received the study supplement starting at least 3 mo prior to conception and continued through delivery; arm 2 started the study supplement at the end of the first trimester and continued through delivery; arm 3 (control) did not receive study supplement. SES, socioeconomic status.

2 *P* values from χ^2^ tests and ANOVA analysis to assess for differences between characteristics by treatment arm.

3 Woman had a live birth in the 24-mo longitudinal analysis subset. After excluding extreme invalid measurements as determined by expert manual review and accounting for biologically implausible *z*-scores based on WHO standards, the 24-mo longitudinal analysis subset included all live-born infants with birth length measurements measured by 7 d (168 h) of age on portable length boards and consented to the offspring follow-up study. Extreme invalid measurements as determined by expert manual review were excluded from the longitudinal analysis. All length-for-age, weight-for-age, weight-for-length, and head-circumference-for-age *z*-scores (LAZ, WAZ, WLZ, and HCAZ, respectively) were calculated using the expanded tables of the Child Growth Standards published by the WHO that provide *z*-scores by sex and age in days at time of measurement. WLZ were calculated using the expanded tables of the Child Growth Standards published by the WHO that provide *z-*scores by sex and tabulated lengths from 45.0 to 110.0 cm. All WHO standards are based on term infants. LAZ, WAZ, WLZ, and HCAZ were within the biologically plausible range according to WHO standards (–6 ≤ LAZ ≤ 6, –6 ≤ WAZ ≤ 6, –5 ≤ WLZ ≤ 5, –5 ≤ HCAZ ≤ 5). If an infant was found to have a biologically implausible LAZ or WAZ according to WHO standards at a visit, all growth outcomes at the visit were set to missing. If an infant was found to have a biologically implausible WLZ or HCAZ according to WHO standards at a visit, only the corresponding measurement and *z*-score at the visit were set to missing. WLZ could not be obtained for infants with a length of <45.0 cm at any visit due to limitations in the WHO standards and were set to missing for that visit (9).

4 This cutoff from the 2007 WHO guidelines (41) was used to reflect stunting for adult women.

5 The SES tally provides the number of indicators available from the following list: electricity, improved water source, sanitation, manmade flooring, improved cooking fuels, and household assets.

### Maternal treatment effects on postnatal longitudinal growth

**FIGURE 1:**
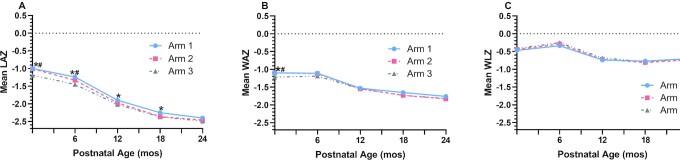
All sites unadjusted longitudinal growth outcomes from birth to 24 mo by treatment arm among the longitudinal analysis subset. Data derived from Supplemental Table 1. *Indicates unadjusted pairwise comparisons between arm 1 compared with arm 3 were significant. ^#^Indicates unadjusted pairwise comparisons between arm 2 compared with arm 3 were significant. Total participants by arms 1, 2, and 3, *n* = 755, 808, and 774, respectively. After excluding extreme invalid measurements as determined by expert manual review and accounting for biologically implausible *z*- scores based on WHO standards (9), the 24-mo longitudinal analysis subset includes all live-born infants with birth length measurements measured by 7 d (168 h) of age on portable length boards and consented to the offspring follow-up study. Arm 1 maternal participants received the study supplement starting at least 3 mo prior to conception and continued through delivery; arm 2 started the study supplement at the end of the first trimester and continued through delivery; arm 3 (control) did not receive study supplement. Sample sizes of offspring according to treatment arm were 755, 808, and 774 for arms 1, 2, and 3, respectively. LAZ, length-for-age *z*-score; WAZ, weight-for-age *z*-score; WLZ, weight-for-length *z*- score.

Means (SDs) of unadjusted anthropometry at 0, 6, 12, and 24 mo and pairwise comparisons among arms are presented in **Supplemental Table 1**. After only modest decline in mean LAZ from birth through 6 mo, the mean LAZ for all arms declined sharply between 6 and 12 mo, with further decline between 12 and 24 mo (**Figure 1**). At birth, 6, 12, and 18 mo, mean LAZ for arm 1 remained significantly greater than the means for arm 3 by pairwise comparisons; mean LAZ for arm 2 was greater than that of arm 3 only at birth and 6 mo. Similar declines, although less steep, were observed for WAZ. The overall rates of stunting progressed from 22.0–30.4% at 6 mo to 62.8%, 64.8%, and 66.3% for arms 1, 2, and 3, respectively, at 24 mo. By site, the mean stunting rates were 73.8%, 65.1%, 74.7%, and 45.5% for the DRC, Guatemala, Pakistan, and India, respectively (Supplemental Table 1 and **Supplemental Figure 2**).

In the adjusted longitudinal models, significant interactions indicating treatment heterogeneity by site were observed for length and LAZ, and site-specific adjusted models were thus used for these 2 outcomes. Significant treatment effects on linear growth were observed only in the DRC (**Supplemental Table 2**), with arm 1 performing better than arms 2 and 3 for both length and LAZ. No other site-specific comparisons among arms demonstrated statistically significant differences for linear growth (Supplemental Table 2). For the combined sites’ longitudinal models, arm 1 compared with 3 was significantly different for weight (*P* = 0.0281), WAZ (*P* = 0.0077), and WLZ (*P* = 0.0337). Separate models that included the interaction effects between arm and time of visit generally demonstrated attenuation of the treatment effects over time (**Supplemental Table 3**).

### Predictors of longitudinal growth trajectory

Determination of demographic predictors of the longitudinal growth trajectory from birth through 24 mo (regardless of any interactions) was the second major objective of this analysis. For LAZ, the strongest predictor was maternal height >150 cm (adjusted mean difference 0.54 SD), followed by paternal height >160 cm, maternal education (secondary), parity ≥1, and male infant sex (negative adjusted mean difference). Treatment arm was a significant predictor of linear growth trajectory, with greater mean difference for both arms 1 and 2 compared with arm 3 (**Table 2**). For WAZ over the same period, significant predictors were similar to those for linear growth. The strongest predictor of WLZ was baseline maternal BMI (>22); maternal education (both primary and secondary) was also significantly and positively associated with WLZ (Table 2).

**TABLE 2:** Predictors in models for longitudinal trajectory for linear and ponderal growth for all infants, all sites^1^

	LAZ		WAZ		WLZ	
Variable	Adjusted mean difference (95% CI)	*P* value	Adjusted mean difference (95% CI)	*P* value	Adjusted mean difference (95% CI)	*P* value
Treatment arm						
Arm 1	0.19 (0.08, 0.30)	0.001	0.11 (0.03, 0.20)	0.009	0.00 (–0.07, 0.07)	0.996
Arm 2	0.17 (0.07, 0.27)	0.001	0.05 (–0.03, 0.13)	0.215	–0.04 (–0.11, 0.04)	0.315
Arm 3	Reference	Reference	Reference	Reference	Reference	Reference
Site						
Guatemala	0.46 (0.03, 0.88)	0.036	0.02 (–0.39, 0.42)	0.931	–0.54 (–0.85, –0.23)	0.001
India	0.56 (0.18, 0.95)	0.004	–0.39 (–0.75, –0.02)	0.038	–1.28 (–1.56, –0.99)	<0.001
Pakistan	0.45 (−0.05, 0.96)	0.077	–0.32 (–0.82, 0.17)	0.201	–1.12 (–1.56, –0.68)	<0.001
Democratic Republic of the Congo	Reference	Reference	Reference	Reference	Reference	Reference
Maternal education						
Primary	0.08 (−0.03, 0.20)	0.152	0.12 (0.01, 0.23)	0.033	0.12 (0.02, 0.21)	0.018
Secondary	0.26 (0.13, 0.39)	<0.001	0.25 (0.12, 0.37)	<0.001	0.13 (0.02, 0.25)	0.018
No formal schooling	Reference	Reference	Reference	Reference	Reference	Reference
Parity						
≥1	0.19 (0.09, 0.29)	<0.001	0.27 (0.17, 0.36)	<0.001	0.17 (0.09, 0.25)	<0.001
0	Reference	Reference	Reference	Reference	Reference	Reference
Tally of indicators of higher SES^2^						
High (3–6 present)	0.11 (0.00, 0.22)	0.043	0.11 (–0.00, 0.21)	0.051	0.05 (–0.04, 0.14)	0.273
Low (0–2 present)	Reference	Reference	Reference	Reference	Reference	Reference
Sex						
Male	–0.13 (–0.20, –0.06)	<0.001	–0.08 (–0.15, –0.01)	0.018	–0.05 (–0.11, 0.01)	0.125
Female	Reference	Reference	Reference	Reference	Reference	Reference
Maternal BMI, kg/m^2^						
>22	0.21 (0.12, 0.29)	<0.001	0.26 (0.17, 0.35)	<0.001	0.19 (0.11, 0.26)	<0.001
≤22	Reference	Reference	Reference	Reference	Reference	Reference
Maternal height, cm						
>150	0.54 (0.46, 0.63)	<0.001	0.39 (0.30, 0.47)	<0.001	0.02 (–0.05, 0.09)	0.638
≤150	Reference	Reference	Reference	Reference	Reference	Reference
Paternal height,^3^ cm						
>160	0.30 (0.21, 0.39)	<0.001	0.20 (0.12, 0.29)	<0.001	0.02 (–0.05, 0.10)	0.565
≤160	Reference	Reference	Reference	Reference	Reference	Reference
Paternal BMI,^3^ kg/m^2^						
>24	0.14 (0.04, 0.23)	0.004	0.16 (0.07, 0.25)	<0.001	0.09 (0.01, 0.17)	0.023
≤24	Reference	Reference	Reference	Reference	Reference	Reference

1 All *z*-scores were calculated using the expanded tables of the Child Growth Standards published by the WHO that provide *z*-scores by sex and age in days at time of measurement. Weight-for-length *z*-scores (WLZ) were calculated using the expanded tables of the Child Growth Standards published by the WHO that provide *z*-scores by sex and tabulated lengths from 45.0 to 110.0 cm. All WHO standards are based on term infants. LAZ, WAZ, and WLZ were within the biologically plausible range according to WHO standards (–6 ≤ LAZ ≤ 6, –6 ≤ WAZ ≤ 6, –5 ≤ WLZ ≤ 5). If an infant was found to have a biologically implausible LAZ or WAZ according to WHO standards at a visit, all growth outcomes at the visit were set to missing. If an infant was found to have a biologically implausible WLZ according to WHO standards at a visit, only the corresponding measurement and *z*-score at the visit were set to missing. WLZ could not be obtained for infants with a length of <45.0 cm at any visit due to limitations in the WHO standards and were set to missing for that visit (9). All the final models included the following predictors: treatment arm; site; cluster; interaction between site and cluster; SES; maternal education, parity, BMI (≤22 and >22), and height (≤150 cm, >150 cm); and paternal BMI (≤22, >22, and missing) and height (≤160 cm, >160 cm, and missing). The interaction between arm and visit was marginally significant (*P* value = 0.067) only for LAZ and was included in the final model; interaction effects are not shown here. Arm 1 maternal participants received the study supplement starting at least 3 mo prior to conception and continued through delivery; arm 2 started the study supplement at the end of the first trimester and continued through delivery; arm 3 (control) did not receive study supplement. Number of infants considered for the combined site analyses according to treatment arm were 755, 808, and 774 for arms 1, 2, and 3, respectively. LAZ, length-for-age *z*-score; SES, socioeconomic status; WAZ, weight-for-age *z-*score; WLZ, weight-for-length *z*-score.

2 The SES tally provides the number of indicators available from the following list: electricity, improved water source, sanitation, manmade flooring, improved cooking fuels, and household assets.

3 Paternal height and BMI had 371 missing records and were included in a separate group: missing group not shown here.

### Predictors of linear growth status at 24 mo

For the third objective, the cross-sectional analyses at 24 mo for the children from all sites indicated the strongest predictors of LAZ were moderate and mild stunting at birth with adjusted mean differences of −0.76 and −0.47 SD, respectively. Maternal height (>150 cm) was associated with adjusted mean difference of +0.45 SD. (**Table 3**). With the inclusion of the birth length in the model, treatment arm was not a significant predictor. Other significant predictors associated with higher adjusted mean difference of LAZ included maternal education (secondary), paternal height, maternal BMI, and higher SES; those associated with lower adjusted mean difference included parity (≥1), LBW, and sex (male) (model *R*^2^ = 0.340). Predictors of stunting, expressed as adjusted RR, included birth LAZ, with both mild and moderate stunting at birth associated with an adjusted RR of 1.38. Maternal height (>150 cm) was associated with an adjusted RR of 0.73; other significant predictors were similar to those for LAZ (Table 3). Correlations among variables that were examined and considered for model selection are presented in **Supplemental Table 4**.

**TABLE 3:** Predictors of LAZ and stunting (LAZ <–2) at 24 mo for all infants, all sites^1^

	LAZ		LAZ <–2	
Variable	Adjusted mean difference (95% CI)	*P* value	Adjusted mean difference (95% CI)	*P* value
Treatment arm				
Arm 1	–0.01 (–0.11, 0.09)	0.796	1.00 (0.92, 1.09)	0.983
Arm 2	–0.04 (–0.14, 0.06)	0.419	1.01 (0.92, 1.10)	0.881
Arm 3	Reference	Reference	Reference	Reference
SES				
High (3–6 present)	0.16 (0.03, 0.28)	0.014	0.94 (0.85, 1.05)	0.288
Low (0–2 present)	Reference	Reference	Reference	Reference
Maternal education				
Primary	0.10 (–0.03, 0.23)	0.14	0.94 (0.85, 1.05)	0.299
Secondary	0.31 (0.16, 0.46)	<0.001	0.79 (0.69, 0.90)	0.001
No formal schooling	Reference	Reference	Reference	Reference
Parity				
≥1	–0.28 (–0.39, –0.17)	<0.001	1.12 (1.02, 1.24)	0.023
0	Reference	Reference	Reference	Reference
Maternal BMI, kg/m^2^				
>22	0.20 (0.10, 0.30)	<0.001	0.90 (0.82, 0.99)	0.031
≤22	Reference	Reference	Reference	Reference
Maternal height, cm				
>150	0.45 (0.36, 0.55)	<0.001	0.73 (0.67, 0.80)	<0.001
≤150	Reference	Reference	Reference	Reference
Paternal height,^2^ cm				
>160	0.26 (0.16, 0.36)	<0.001	0.85 (0.77, 0.92)	<0.001
≤160	Reference	Reference	Reference	Reference
Paternal BMI^3^				
>24	0.08 (–0.03, 0.18)	0.15	0.96 (0.87, 1.06)	0.45
≤24	Reference	Reference	Reference	Reference
Birth stunting				
Mild (<–1 to ≥–2)	–0.47 (–0.56, –0.38)	<0.001	1.38 (1.27, 1.49)	<0.001
Moderate (LAZ <–2)	–0.76 (–0.93, –0.58)	<0.001	1.38 (1.20, 1.60)	<0.001
None (LAZ ≥–1)	Reference	Reference	Reference	Reference
Birth WLZ <–2				
No	–0.11 (–0.28, 0.07)	0.236	1.10 (0.93, 1.29)	0.27
Yes	Reference	Reference	Reference	Reference
Sex				
Male	–0.13 (–0.21, –0.05)	0.001	1.10 (1.02, 1.18)	0.01
Female	Reference	Reference	Reference	Reference
Low birth weight				
Yes	–0.24 (–0.38, –0.10)	0.001	1.16 (1.04, 1.30)	0.01
No	Reference	Reference	Reference	Reference

1 All *z*-scores were calculated using the expanded tables of the Child Growth Standards published by the WHO that provide *z*-scores by sex and age in days at time of measurement. WLZ were calculated using the expanded tables of the Child Growth Standards published by the WHO that provide *z*-scores by sex and tabulated lengths from 45.0 to 110.0 cm. All WHO standards are based on term infants. LAZ, WAZ, and WLZ were within the biologically plausible range according to WHO standards (–6 ≤ LAZ ≤ 6, –6 ≤ WAZ ≤ 6, –5 ≤ WLZ ≤ 5). If an infant was found to have a biologically implausible LAZ or WAZ according to WHO standards at a visit, all growth outcomes at the visit were set to missing. If an infant was found to have a biologically implausible WLZ according to WHO standards at a visit, only the corresponding measurement and *z-*score at the visit were set to missing. WLZ could not be obtained for infants with a length of <45.0 cm at any visit due to limitations in the WHO standards and were set to missing for that visit (9). All the final models included the following predictors: arm; site; cluster; interaction between site and cluster; SES; maternal education, parity, BMI (≤22 and >22), and height (≤150 cm, >150 cm); and paternal BMI (≤22, >22, and missing) and height (≤160 cm, >160 cm, and missing); birth LAZ <–1; birth LAZ <–2; birth WLZ <–2; infant sex; and low birth weight. Arm 1 maternal participants received the study supplement starting at least 3 mo prior to conception and continued through delivery; arm 2 started the study supplement at the end of the first trimester and continued through delivery; arm 3 (control) did not receive study supplement. Number of infants considered for the combined site analysis according to treatment arm were 713, 773, and 735 for arms 1, 2, and 3, respectively. LAZ, length-for-age *z*-score; SES, socioeconomic status; WAZ, weight-for-age *z*-score; WLZ, weight-for-length *z*-score.

2 Model *R*^2^ = 0.340.

3 Paternal height and BMI had 371 missing records and were included in a separate group: missing group not shown here.

**FIGURE 2:**
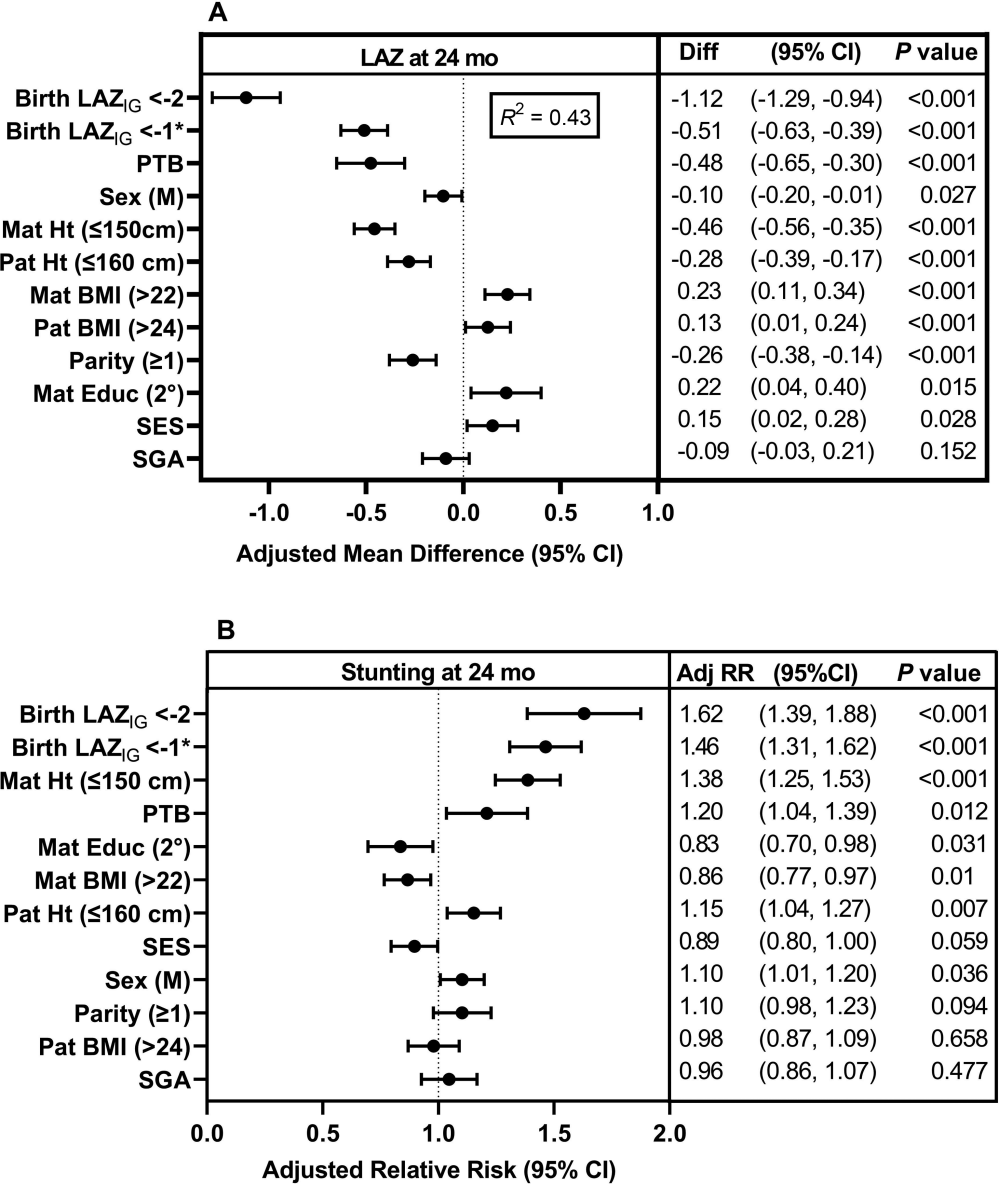
Predictors of length-for-age *z*-score (LAZ) (2A) and stunting (2B) at 24 mo for infants with gestational age determined by first-trimester ultrasound. Cross-sectional analyses with linear (robust Poisson) regression for continuous (binary) outcomes were used to identify the major predictors of linear growth status at 24 mo. Gestational age was determined by first-trimester ultrasound and *z*-scores were derived from INTERGROWTH-21st fetal growth charts (10). Total participants *n* = 1329; by arms 1, 2, 3: *n* = 438, 478, 413, respectively; by site: Guatemala *n* = 439, India *n* = 487, Pakistan *n* = 403. Model included adjustment for arm, site, cluster, and interaction between site and cluster. *Birth LAZ <–1 to ≥–2. Adj RR, adjusted RR; Educ, education; Ht, height; IG, INTERGROWTH-21st standards; LAZ, length-for-age *z*-score; Mat, maternal; Pat, paternal; PTB, preterm birth; SES, socioeconomic status; SGA, small for gestational age.

For the subgroup of infants with gestational age determinations, the strongest predictors of LAZ at 24 mo were also stunting at birth (LAZ _IG_ <–2 and <–1 to ≥–2), each associated with significant and negative adjusted mean differences of –1.12 SD and –0.51 SD, respectively; preterm birth was associated with an adjusted mean difference of −0.48 SD. Maternal and paternal heights (≤150 and ≤160 cm, respectively) were negatively associated, whereas maternal BMI, maternal education (secondary), and SES were positively associated with adjusted mean differences (model *R*^2^ = 0.432) (**Figure 2**A). The strongest predictors of stunting were moderate and mild stunting at birth, with adjusted RR of 1.62 and 1.46 , respectively (Figure 2B). Low parental heights and preterm birth were also associated with an increased risk of stunting, with RR between 1.15 to 1.38. Maternal education and BMI >22 were significant predictors of lower risk of stunting (Figure 2B). Correlations among variables that were examined and considered for model selection are presented in **Supplemental Table 5**.

## Discussion

The most striking findings of the present analysis of growth status at 2 y of the children of participants in the WF trial were the severe stunting rates; evidence of persistence of a treatment effect on longitudinal linear growth for the preconception arm, which was driven predominantly by one site (DRC); that both maternal and paternal heights were the strongest predictors of longitudinal linear growth trajectory; and that deficits in birth length were the strongest predictors of attained LAZ and of stunting at 2 y. With no postnatal intervention, infants in both maternal intervention arms experienced a steep decline in LAZ between 6 and 12 mo and a slower but steady downward progression thereafter. Mild and moderate stunting at birth were associated with 0.5 to 1.0 lower adjusted mean difference in LAZ and a 40–60% increase in stunting risk at 2 y. Thus, despite the overall pattern of linear growth faltering for the offspring of mothers in all arms of the primary trial (1), a smaller length deficit at birth was associated with a persistent beneficial impact on linear growth.

The profound stunting rates at 2 y contrast with the steady decline in global stunting reported in recent analyses, which have indicated overall declines to ∼20%, although with substantial variability among and within countries (5, 6, 11). The data from the WF sites underscore this variability, with rates of ∼65% for the offspring of women in 3 of the study sites and with the Indian site having a mean rate of 45%. As recently highlighted (12, 13), the cut-point of 2 SD below the median to define stunting should not obscure the reality that the observed mean (and median) LAZ of –2.4 at 24 mo in the WF offspring portends a high risk for loss of developmental potential for a very large percentage of the young children in these settings. The pattern of linear growth faltering we observed indicates little improvement or even worsening over the past decade for 3 of the WF sites (DRC, Guatemala, and Pakistan), each of which participated in a complementary feeding trial a decade ago and were observed to have stunting rates of ∼50% at 18 mo (14). A recent analysis of drivers of stunting found that non–health sector factors accounted for nearly 50% of improvements in stunting, with improvements in wealth, maternal education, and urbanization all being associated with lower stunting rates (15). In contrast, the WF participants bore the hallmarks of stunting risk—impoverished, undernourished, and poorly educated—and our results convincingly reinforce the need for multipronged interventions to reduce stunting.

A major incentive for conducting this follow-up analysis was to determine the potential for persistence of the benefits to fetal growth of the preconception and early pregnancy nutrition intervention on postnatal growth, without any additional interventions. Although the intervention effects on both linear and ponderal growth were evident through 6 mo (2), these follow-up data strongly argue for continued attention to the remainder of the 1000 d, including complementary feeding. Qualitative feeding evaluations of the WF offspring indicated continued breastfeeding but inadequate complementary feeding for the majority of the infants, especially during the crucial period between 6 and 12 mo, when energy and nutrient needs remain relatively high and when we observed the sharpest decline in LAZ (16). However, nutrition interventions initiated after 6 mo of age have generally had a modest, if any, impact on linear growth or rates of stunting in the second year of life (14, 17–23). In addition, recent intensive interventions to improve water, sanitation, and hygiene in settings with high stunting rates had virtually no impact on postnatal linear growth (24). These intervention results notwithstanding, our observations reinforce the importance of improving the infant and young child's nutrition and health environment. Absent such attention, gains realized in fetal growth from early maternal interventions seem unlikely to persist past the early postnatal months.

The analyses for the longitudinal trajectory across the entire study period identified parental heights (especially maternal) and intervention arm of the primary trial as significant predictors. However, with inclusion of birth length in the cross-sectional analysis at 24 mo, the intervention arm was no longer significant, and birth length outcomes were the strongest predictors of lower LAZ and risk of stunting; for the group of infants with gestational age determinations, moderate stunting at birth had the highest risk of stunting at 24 mo. Low birth weight (but not SGA) was a significant predictor but had a considerably smaller impact on the adjusted mean difference in LAZ at 24 mo compared with those associated with the deficits in birth length, supporting the premise that very early fetal growth may program longer-term linear growth (3, 25). Our observations complement those for a large cohort of infants in Zimbabwe in whom birth length status, along with maternal height and education and other factors, was associated with the pattern and degree of postnatal linear growth faltering (26).

Among predictive factors, in this analysis and numerous other studies, maternal height was a robust predictor of the longitudinal linear growth trajectory and of LAZ and stunting at 2 y (21, 26–29), with height <150 cm associated with a 38% increase in stunting risk. Maternal height and pelvic dimensions affect placental dimensions and function, including nutrient transfer, and are associated with newborn size, including birth length (30) as well as risk of future chronic conditions (31). In a subgroup of the WF participants in Pakistan, a high percentage of whom were stunted and thin, women who received preconception and first-trimester SQ-LNS (intervention arm 1) had a significantly larger (+1.4-fold, *P* = 0.03) placental area than the control arm, and placental area was correlated with LAZ at birth (32). This suggests that maternal nutrition during the first trimester is a modifiable factor with potential to attenuate the growth-restraining effects of maternal short stature. Compared with maternal height, fewer data on the impact of paternal height are available, but it is notable that in our study, both paternal height and BMI were significantly associated with LAZ and stunting at 24 mo. These observations support the increasing recognition of adolescence as a nutrition-sensitive developmental period and a window of opportunity to favorably affect adult height for both women and men, thereby potentially improving reproductive outcomes (33, 34).

Emphasis on maternal factors is emerging as a critical and potentially modifiable determinant of child stunting (21), particularly when improvements in women's nutrition and overall health and in environmental factors occur prior to conception (35). Although both the preconception and end of first trimester arms of the WF trial demonstrated improved birth length, several indicators support a greater benefit of the preconception intervention (compared with no intervention arm) in infants with gestational age determination, including significantly reduced risk for moderate stunting and wasting at birth only in the preconception arm (1, 36); a larger impact on both length and weight at birth of the preconception arm for nulliparous and anemic women at enrollment (37); and optimized maternal weight status (associated with the protein energy supplement) prior to conception and greater gestational weight gain before 12 wk of gestation (38–40). These findings provide targets for interventions to improve fetal growth and thereby to potentially improve subsequent growth during the first 2 y of life. Last, we highlight the finding that maternal education (at least secondary) emerged as a consistent and modifiable predictor of improved longitudinal growth (linear and ponderal) and reduced stunting at 24 mo (5, 7).

The strengths of this analysis include the high percentage (>95%) of offspring of the WF trial participants who contributed to the data through 24 mo and supported a rigorous analysis of longitudinal postnatal growth. The multicountry design of the original randomized trial is a strength. Although the results reflect heterogeneous settings and participants, these features also support generalizability of the findings. Limitations include the availability of first-trimester gestational age dating from only 3 of the 4 sites and the absence of data on postnatal morbidity and biochemical indicators of inflammatory, metabolic, and nutritional status that could potentially have provided insights into factors driving the linear growth faltering we observed. However, given the strength of the predictive variables and their consistency with observations from the literature, we submit that the impact of such postnatal factors is unlikely to have substantially attenuated the findings.

In summary, the sharp downward trajectory of linear growth after 6 mo of age and the profound stunting rates at 24 mo underscore the importance of postnatal nutrition (and other environmental/nutrition-sensitive factors), which surely must be addressed. The evidence to date, however, suggests the impact of such interventions will be incremental without attention to the entire 1000 d. More research is needed to determine whether additive, or even synergistic, effects could be realized with a combination of early prenatal and postnatal interventions. With the strongest predictor of stunting (and length) at 24 mo being birth length, however, we surmise that substantial improvements in postnatal growth are likely to depend on improved intrauterine growth, especially during the critical first trimester.

## Supplementary Material

nqac051_Supplemental_File

## Data Availability

Deidentified study data will be available through the NICHD Data and Specimen Hub at https://dash.nichd.nih.gov upon publication.

## Notes

Supported by the Bill & Melinda Gates Foundation (OPP1055867) and the *Eunice Kennedy Shriver* National Institute of Child Health and Human Development and the Office of Dietary Supplements (NIH U10 HD 076474). The Bill & Melinda Gates Foundation had no role in study design, data collection, data analysis, data interpretation, or writing of this report. National Institute of Child Health and Human Development (NICHD) scientists provided input into study conduct and content of the manuscript. The content is solely the responsibility of the authors and does not necessarily represent the official view of the NIH.

Supplemental Figures 1–2 and Supplemental Tables 1–5 are available from the “Supplementary data” link in the online posting of the article and from the same link in the online table of contents at https://academic.oup.com/ajcn/.

Abbreviations used: DCC, data coordinating center; DRC, Democratic Republic of the Congo; GEE, generalized estimating equation; HVRA, home visitor research assistant; IG, INTERGROWTH-21st; LAZ, length-for-age *z*-score; LWB, low birth weight; SES, socioeconomic status; SGA, small for gestational age; SQ-LNS, small-quantity lipid nutrient supplement; WAZ, weight-for-age *z-*score; WF, Women First; WLZ, weight-for-length *z*-score.
