# Supplementary material for: Birth length is the strongest predictor of linear growth status and stunting in the first 2 years of life after a preconception maternal nutrition intervention: the children of the Women First trial
Source: Am J Clin Nutr. 2022 Jun 10;116(1):86–96. doi: 10.1093/ajcn/nqac051 (PMC9257468; doi:10.1093/ajcn/nqac051)
Supplement: nqac051_Supplemental_File [file nqac051_supplemental_file.pdf]

**Birth length is the strongest predictor of linear growth status and stunting in the first 2 years of life after a preconception maternal nutrition intervention: the children of the Women First trial (Krebs, et al)**

**Online Supplementary Material**

**Supplemental Table 1.** All sites unadjusted growth outcomes from birth to 24 months by treatment arm among the longitudinal analysis subset [1]

| | | | | Unadjusted Pairwise Arm Comparisons and 95% CI [3] | | |
|---|---|---|---|---|---|---|
| **Variable [2]** | **Arm 1 (N=755)** | **Arm 2 (N=808)** | **Arm 3 (N=774)** | **Arm 1 vs. 3** | **Arm 2 vs. 3** | **Arm 1 vs. 2** |
| **Continuous Variables** | **Mean (n, SD)** | **Mean (n, SD)** | **Mean (n, SD)** | **Mean Difference (95% CI)** | **Mean Difference (95% CI)** | **Mean Difference (95% CI)** |
| Length, cm | | | | | | |
| Birth (<7 days) | 47.65 (755, 2.18) | 47.63 (808, 2.03) | 47.32 (774, 2.09) | 0.34 (0.12, 0.55) | 0.31 (0.11, 0.51) | 0.03 (-0.18, 0.24) |
| 6 months | 63.85 (747, 2.58) | 63.62 (801, 2.69) | 63.41 (769, 2.65) | 0.44 (0.18, 0.71) | 0.21 (-0.05, 0.47) | 0.23 (-0.03, 0.50) |
| 12 months | 70.20 (730, 3.05) | 69.98 (797, 3.09) | 69.92 (752, 2.94) | 0.27 (-0.03, 0.58) | 0.06 (-0.24, 0.36) | 0.22 (-0.09, 0.52) |
| 18 months | 75.18 (724, 3.37) | 74.83 (789, 3.41) | 74.86 (746, 3.42) | 0.32 (-0.03, 0.67) | -0.03 (-0.37, 0.31) | 0.35 (0.01, 0.69) |
| 24 months | 79.18 (712, 3.70) | 78.96 (772, 3.74) | 78.92 (735, 3.60) | 0.26 (-0.12, 0.64) | 0.04 (-0.33, 0.41) | 0.22 (-0.16, 0.60) |
| Length-for-age Z-score (LAZ) | | | | | | |
| Birth (<7 days) | -1.00 (755, 1.15) | -1.01 (808, 1.07) | -1.19 (774, 1.10) | 0.19 (0.07, 0.30) | 0.17 (0.07, 0.28) | 0.01 (-0.10, 0.12) |
| 6 months | -1.24 (747, 1.12) | -1.33 (801, 1.18) | -1.45 (769, 1.16) | 0.21 (0.10, 0.33) | 0.12 (0.00, 0.23) | 0.10 (-0.02, 0.21) |
| 12 months | -1.90 (730, 1.19) | -1.97 (797, 1.21) | -2.02 (752, 1.16) | 0.12 (0.00, 0.24) | 0.05 (-0.07, 0.17) | 0.07 (-0.05, 0.19) |
| 18 months | -2.25 (724, 1.17) | -2.36 (789, 1.19) | -2.37 (746, 1.20) | 0.13 (0.01, 0.25) | 0.02 (-0.10, 0.14) | 0.11 (-0.01, 0.23) |
| 24 months | -2.40 (712, 1.15) | -2.46 (772, 1.17) | -2.49 (735, 1.13) | 0.09 (-0.03, 0.21) | 0.03 (-0.09, 0.15) | 0.06 (-0.06, 0.18) |
| Weight, gm | | | | | | |
| Birth (<7 days) | 2812 (755, 433) | 2812 (808, 412) | 2763 (774, 414) | 48.9 (6.3, 91.4) | 48.7 (8.0, 90.0) | 0.1 (-41.8, 42.1) |
| 6 months | 6743 (749, 1048) | 6725 (804, 1031) | 6684 (770, 1018) | 59.3 (-44.7, 163.3) | 40.6 (-60.8, 142.0) | 18.7 (-84.8, 122.2) |
| 12 months | 7878 (729, 1143) | 7843 (798, 1119) | 7866 (750, 1110) | 12.1 (-102.8, 127.0) | -22.3 (-133.5, 88.9) | 34.4 (-79.3, 148.0) |
| 18 months | 8819. (724, 1198) | 8719 (788, 1185) | 8753 (745, 1186) | 66.5 (-55.5, 188.6) | -33.4 (-152.2, 85.4) | 99.9 (-20.4, 220.2) |
| 24 months | 9696 (712, 1300) | 9602 (773, 1278) | 9632 (735, 1249) | 65.0 (-66.5, 196.4) | -29.0 (-156.8, 98.7) | 94.0 (-37.3, 225.3) |

| Variable [2] | Arm 1 (N=755) | Arm 2 (N=808) | Arm 3 (N=774) | Unadjusted Pairwise Arm Comparisons and 95% CI [3] Arm 1 vs. 3 | Arm 2 vs. 3 | Arm 1 vs. 2 |
|---|---|---|---|---|---|---|
| **Continuous Variables** | **Mean (n, SD)** | **Mean (n, SD)** | **Mean (n, SD)** | **Mean Difference (95% CI)** | **Mean Difference (95% CI)** | **Mean Difference (95% CI)** |
| Length, cm | | | | | | |
| Weight-for-age Z-score (WAZ) | | | | | | |
| Birth (<7 days) | -1.10 (755, 1.02) | -1.10 (808, 0.97) | -1.22 (774, 0.98) | 0.11 (0.01, 0.22) | 0.12 (0.02, 0.22) | -0.01 (-0.11, 0.09) |
| 6 months | -1.11 (749, 1.31) | -1.12 (804, 1.30) | -1.19 (770, 1.27) | 0.08 (-0.05, 0.21) | 0.07 (-0.06, 0.20) | 0.01 (-0.12, 0.14) |
| 12 months | -1.53 (729, 1.23) | -1.55 (798, 1.21) | -1.55 (750, 1.20) | 0.02 (-0.10, 0.14) | -0.00 (-0.12, 0.12) | 0.02 (-0.10, 0.15) |
| 18 months | -1.65 (724, 1.17) | -1.73 (788, 1.16) | -1.73 (745, 1.14) | 0.08 (-0.04, 0.20) | -0.01 (-0.12, 0.11) | 0.09 (-0.03, 0.20) |
| 24 months | -1.76 (712, 1.12) | -1.83 (773, 1.12) | -1.82 (735, 1.09) | 0.06 (-0.06, 0.17) | -0.01 (-0.12, 0.10) | 0.07 (-0.04, 0.19) |
| Weight-for-length Z-score (WLZ) | | | | | | |
| Birth (<7 days) | -0.47 (675, 1.06) | -0.44 (729, 1.05) | -0.42 (675, 0.99) | -0.05 (-0.16, 0.06) | -0.03 (-0.13, 0.08) | -0.03 (-0.14, 0.08) |
| 6 months | -0.34 (745, 1.27) | -0.29 (801, 1.25) | -0.25 (767, 1.20) | -0.09 (-0.21, 0.03) | -0.03 (-0.15, 0.09) | -0.06 (-0.18, 0.07) |
| 12 months | -0.74 (728, 1.16) | -0.72 (797, 1.15) | -0.68 (749, 1.15) | -0.06 (-0.18, 0.05) | -0.05 (-0.16, 0.07) | -0.02 (-0.13, 0.10) |
| 18 months | -0.77 (722, 1.11) | -0.81 (788, 1.12) | -0.79 (744, 1.09) | 0.03 (-0.09, 0.14) | -0.02 (-0.13, 0.09) | 0.04 (-0.07, 0.16) |
| 24 months | -0.70 (712, 1.08) | -0.74 (772, 1.07) | -0.71 (735, 1.06) | 0.01 (-0.10, 0.12) | -0.03 (-0.14, 0.08) | 0.04 (-0.07, 0.15) |
| Head Circumference, cm | | | | | | |
| Birth (<7 days) | 33.3 (751, 1.5) | 33.3 (805, 1.4) | 33.2 (772, 1.5) | 0.03 (-0.11, 0.18) | 0.05 (-0.09, 0.19) | -0.01 (-0.15, 0.13) |
| 6 months | 41.5 (743, 1.6) | 41.5 (802, 1.5) | 41.5 (767, 1.5) | -0.02 (-0.18, 0.14) | -0.05 (-0.20, 0.10) | 0.02 (-0.13, 0.18) |
| 12 months | 44.0 (727, 1.6) | 43.9 (795, 1.5) | 44.0 (747, 1.5) | -0.04 (-0.20, 0.12) | -0.07 (-0.22, 0.08) | 0.03 (-0.12, 0.19) |
| 18 months | 45.2 (722, 1.6) | 45.2 (785, 1.5) | 45.2 (742, 1.5) | -0.04 (-0.19, 0.12) | -0.07 (-0.22, 0.07) | 0.04 (-0.12, 0.19) |
| 24 months | 46.1 (711, 1.6) | 46.0 (769, 1.4) | 46.1 (733, 1.5) | -0.02 (-0.18, 0.13) | -0.05 (-0.19, 0.10) | 0.03 (-0.13, 0.18) |
| Head Circumference-for-age Z-score (HCAZ) | | | | | | |
| Birth (<7 days) | -0.76 (751, 1.16) | -0.74 (805, 1.09) | -0.79 (772, 1.16) | 0.03 (-0.08, 0.15) | 0.05 (-0.06, 0.16) | -0.02 (-0.13, 0.10) |
| 6 months | -0.96 (743, 1.20) | -0.97 (802, 1.12) | -0.95 (767, 1.14) | -0.01 (-0.13, 0.11) | -0.02 (-0.13, 0.09) | 0.01 (-0.11, 0.12) |
| 12 months | -1.16 (727, 1.10) | -1.17 (795, 1.06) | -1.14 (747, 1.06) | -0.03 (-0.14, 0.08) | -0.03 (-0.14, 0.07) | 0.01 (-0.10, 0.12) |

| Variable [2] | Arm 1 (N=755) | Arm 2 (N=808) | Arm 3 (N=774) | Unadjusted Pairwise Arm Comparisons and 95% CI [3] Arm 1 vs. 3 | Arm 2 vs. 3 | Arm 1 vs. 2 |
|---|---|---|---|---|---|---|
| **Continuous Variables** | **Mean (n, SD)** | **Mean (n, SD)** | **Mean (n, SD)** | **Mean Difference (95% CI)** | **Mean Difference (95% CI)** | **Mean Difference (95% CI)** |
| Length, cm | | | | | | |
| 18 months | -1.19 (722, 1.08) | -1.20 (785, 1.02) | -1.17 (742, 1.01) | -0.02 (-0.12, 0.09) | -0.03 (-0.13, 0.07) | 0.01 (-0.09, 0.12) |
| 24 months | -1.21 (711, 1.05) | -1.21 (769, 0.99) | -1.20 (733, 0.98) | -0.01 (-0.11, 0.10) | -0.01 (-0.11, 0.09) | 0.01 (-0.10, 0.11) |
| **Dichotomous Variables** | **n/N (%)** | **n/N (%)** | **n/N (%)** | **Relative Risk (95% CI)** | **Relative Risk (95% CI)** | **Relative Risk (95% CI)** |
| LAZ <-2 | | | | | | |
| Birth (<7 days) | 123/755 (16.3) | 121/808 (15.0) | 168/774 (21.7) | 0.75 (0.61, 0.93) | 0.69 (0.56, 0.85) | 1.09 (0.86, 1.37) |
| 6 months | 171/747 (22.9) | 203/801 (25.3) | 234/769 (30.4) | 0.75 (0.63, 0.89) | 0.83 (0.71, 0.98) | 0.90 (0.76, 1.08) |
| 12 months | 329/730 (45.1) | 383/797 (48.1) | 369/752 (49.1) | 0.92 (0.82, 1.02) | 0.98 (0.88, 1.09) | 0.94 (0.84, 1.04) |
| 18 months | 419/724 (57.9) | 476/789 (60.3) | 463/746 (62.1) | 0.93 (0.86, 1.01) | 0.97 (0.90, 1.05) | 0.96 (0.88, 1.04) |
| 24 months | 447/712 (62.8) | 500/772 (64.8) | 487/735 (66.3) | 0.95 (0.88, 1.02) | 0.98 (0.91, 1.05) | 0.97 (0.90, 1.05) |
| WAZ <-2 | | | | | | |
| Birth (<7 days) | 127/755 (16.8) | 131/808 (16.2) | 147/774 (19.0) | 0.89 (0.71, 1.10) | 0.85 (0.69, 1.06) | 1.04 (0.83, 1.30) |
| 6 months | 165/749 (22.0) | 169/804 (21.0) | 183/770 (23.8) | 0.93 (0.77, 1.12) | 0.88 (0.74, 1.06) | 1.05 (0.87, 1.27) |
| 12 months | 234/729 (32.1) | 257/798 (32.2) | 251/750 (33.5) | 0.96 (0.83, 1.11) | 0.96 (0.83, 1.11) | 1.00 (0.86, 1.15) |
| 18 months | 255/724 (35.2) | 311/788 (39.5) | 300/745 (40.3) | 0.87 (0.77, 1.00) | 0.98 (0.87, 1.11) | 0.89 (0.78, 1.02) |
| 24 months | 280/712 (39.3) | 323/773 (41.8) | 310/735 (42.2) | 0.93 (0.82, 1.06) | 0.99 (0.88, 1.12) | 0.94 (0.83, 1.06) |
| WLZ <-2 | | | | | | |
| Birth (<7 days) | 50/675 (7.4) | 51/729 (7.0) | 36/675 (5.3) | 1.39 (0.92, 2.10) | 1.31 (0.87, 1.98) | 1.06 (0.73, 1.54) |
| 6 months | 64/745 (8.6) | 74/801 (9.2) | 58/767 (7.6) | 1.14 (0.81, 1.60) | 1.22 (0.88, 1.70) | 0.93 (0.68, 1.28) |
| 12 months | 101/728 (13.9) | 103/797 (12.9) | 91/749 (12.1) | 1.14 (0.88, 1.49) | 1.06 (0.82, 1.38) | 1.07 (0.83, 1.39) |

| | | | | | | |
|---|---|---|---|---|---|---|
| 18 months | 97/722 (13.4) | 101/788 (12.8) | 93/744 (12.5) | 1.07 (0.82, 1.40) | 1.03 (0.79, 1.33) | 1.05 (0.81, 1.36) |
| 24 months | 73/712 (10.3) | 90/772 (11.7) | 74/735 (10.1) | 1.02 (0.75, 1.38) | 1.16 (0.87, 1.55) | 0.88 (0.66, 1.18) |

[1] After excluding extreme invalid measurements as determined by expert manual review and accounting for biologically implausible Z-scores based on WHO standards [b], the 24-month longitudinal analysis subset included all live born infants with birth length measurements measured by 7 days (168 hours) of age on portable infantometers/pediatric stadiometers, and consented to the offspring follow-up study.

[2] Extreme invalid measurements as determined by expert manual review were excluded from the longitudinal analysis. All length-for-age, weight-for-age, weight-for-length, and head-circumference-for-age Z-scores (LAZ, WAZ, WLZ, and HCAZ, respectively) were calculated using the expanded tables of the Child Growth Standards published by the WHO that provide Z-scores by sex and age in days at time of measurement. Weight-for-length Z-scores (WLZ) were calculated using the expanded tables of the Child Growth Standards published by the WHO that provide Z-scores by sex and tabulated lengths from 45.0 cm to 110.0 cm. All WHO standards are based on term infants. LAZ, WAZ, WLZ, and HCAZ were within the biologically plausible range according to WHO standards ($-6 \leq LAZ \leq 6$, $-6 \leq WAZ \leq 6$, $-5 \leq WLZ \leq 5$, $-5 \leq HCAZ \leq 5$). If an infant was found to have a biologically implausible LAZ or WAZ according to WHO standards at a visit, all growth outcomes at the visit were set to missing. If an infant was found to have a biologically implausible WLZ or HCAZ according to WHO standards at a visit, only the corresponding measurement and Z-score at the visit were set to missing. WLZ could not be obtained for infants with a length of less than 45.0 cm at any visit due to limitations in the WHO standards and were set to missing for that visit (1).

[3] Unadjusted pairwise mean differences and 95% confidence limits (CI) were obtained from t-test assuming equal variance across arms.

Arm 1 maternal participants received the study supplement starting at least 3 months prior to conception and continued through delivery; Arm 2 started the study supplement at the end of the first trimester and continued through delivery; Arm 3 (Control) did not receive study supplement. Number of infants considered for combined site analyses was 755, 808, and 774 in Arms 1, 2, and 3, respectively.

**Birth length is the strongest predictor of linear growth status and stunting in the first 2 years of life after a preconception maternal nutrition intervention: the children of the Women First trial (Krebs, et al)**

**Online Supplementary Material**

**Supplemental Table 2.** Effect sizes and relative risks for 0-24 month continuous and categorical anthropometric outcomes, respectively, for the longitudinal analysis [1]

| Variable | Arm 1 vs. Arm 3 | | Arm 2 vs. Arm 3 | | Arm 1 vs. Arm 2 | |
|---|---|---|---|---|---|---|
| **Continuous Variables [2]** | **Effect Size (95% CI)** | ***P* value** | **Effect Size (95% CI)** | ***P* value** | **Effect Size (95% CI)** | ***P* value** |
| Length, cm [3] | | | | | | |
| Democratic Republic of Congo | 0.84 (0.41, 1.27) | 0.0001 | 0.29 (-0.12, 0.69) | 0.162 | 0.55 (0.15, 0.96) | 0.0075 |
| Guatemala | -0.06 (-0.44, 0.32) | 0.756 | 0.11 (-0.28, 0.49) | 0.591 | -0.17 (-0.57, 0.23) | 0.416 |
| India | 0.31 (-0.12, 0.74) | 0.164 | 0.22 (-0.21, 0.65) | 0.318 | 0.09 (-0.36, 0.53) | 0.706 |
| Pakistan | 0.30 (-0.17, 0.78) | 0.214 | 0.12 (-0.34, 0.58) | 0.607 | 0.18 (-0.26, 0.62) | 0.423 |
| Length-for-age Z-score (LAZ) [4] | | | | | | |
| Democratic Republic of Congo | 0.36 (0.18, 0.54) | <0.0001 | 0.14 (-0.03, 0.31) | 0.098 | 0.22 (0.05, 0.39) | 0.0101 |
| Guatemala | -0.03 (-0.19, 0.13) | 0.702 | 0.06 (-0.09, 0.22) | 0.444 | -0.09 (-0.25, 0.07) | 0.272 |
| India | 0.13 (-0.04, 0.31) | 0.141 | 0.10 (-0.07, 0.28) | 0.247 | 0.03 (-0.15, 0.21) | 0.759 |
| Pakistan | 0.17 (-0.02, 0.37) | 0.084 | 0.08 (-0.11, 0.26) | 0.427 | 0.10 (-0.09, 0.28) | 0.301 |
| Weight (gm) | 78.3 (8.4, 148.1) | 0.0281 | 15.4 (-51.9, 82.8) | 0.653 | 62.8 (-5.7, 131.4) | 0.072 |
| Weight-for-age Z-score (WAZ) | 0.12 (0.03, 0.20) | 0.0077 | 0.05 (-0.03, 0.13) | 0.254 | 0.07 (-0.01, 0.15) | 0.107 |
| Weight-for-length. kg/m | 0.09 (0.01, 0.17) | 0.0337 | 0.02 (-0.06, 0.09) | 0.693 | 0.07 (-0.01, 0.15) | 0.074 |
| Weight-for-length Z-score (WLZ) | 0.00 (-0.07, 0.08) | 0.920 | -0.04 (-0.11, 0.03) | 0.294 | 0.04 (-0.03, 0.12) | 0.263 |
| **Dichotomous Variables** | **Relative Risk (95% CI)** | **p value** | **Relative Risk (95% CI)** | **p value** | **Relative Risk (95% CI)** | **p value** |
| LAZ <-2 [5] | 0.94 (0.88, 1.01) | 0.081 | 0.98 (0.92, 1.04) | 0.515 | 0.96 (0.90, 1.03) | 0.245 |
| WAZ <-2 [6] | 0.90 (0.81, 0.99) | 0.0338 | 0.96 (0.88, 1.06) | 0.441 | 0.93 (0.84, 1.03) | 0.172 |
| WLZ <-2 [7] | 1.02 (0.86, 1.21) | 0.822 | 1.08 (0.92, 1.28) | 0.348 | 0.94 (0.80, 1.11) | 0.476 |

[1] After excluding extreme invalid measurements as determined by expert manual review [8], the longitudinal analysis subset includes all live born infants with birth length measurements measured by 7 days (168 hours) of age on portable length boards, and had consented to participate in the follow-up study.

[2] Using longitudinal generalized linear models with generalized estimating equations (GEE), assuming a normal distribution and the identity link, and Auto-regressive order 1 covariance structure. Adjusted for site, cluster nested within site, visit, maternal education, stunting, parity, socioeconomic status (SES), and infant sex.

[3] In the longitudinal analysis of length, the interaction between intervention and site was significant at $\alpha=0.10$ (*P* value=0.0972), and thus site specific estimates are presented.

[4] In the longitudinal analysis of length-for-age Z-scores (LAZ), the interaction between intervention and site was significant at $\alpha=0.10$ (*P* value=0.0620), and thus site specific estimates are presented.

[5] Using longitudinal generalized linear models with GEE, assuming a binary distribution and the log link, and Auto-regressive order 1 covariance structure. Adjusted for site, visit, maternal education, parity, SES, and infant sex.

[6] Using longitudinal generalized linear models with GEE, assuming a binary distribution and the log link, and Auto-regressive order 1 covariance structure. Adjusted for site, cluster nested within site, visit, maternal education, parity, SES, and infant sex.

[7] Using longitudinal generalized linear models with GEE, assuming a binary distribution and the log link, and Auto-regressive order 1 covariance structure. Adjusted for site, visit, maternal education, stunting, parity, SES, and infant sex.

[8] All Z-scores were calculated using the expanded tables of the Child Growth Standards published by the WHO that provide Z-scores by sex and age in days at time of measurement. Weight-for-length Z-scores (WLZ) were calculated using the expanded tables of the Child Growth Standards published by the WHO that provide Z-scores by sex and tabulated lengths from 45.0 cm to 110.0 cm. All WHO standards are based on term infants. LAZ, WAZ, and WLZ were within the biologically plausible range according to WHO standards ($-6 \leq LAZ \leq 6$, $-6 \leq WAZ \leq 6$, $-5 \leq WLZ \leq 5$). If an infant was found to have a biologically implausible LAZ or WAZ according to WHO standards at a visit, all growth outcomes at the visit were set to missing. If an infant was found to have a biologically implausible WLZ according to WHO standards at a visit, only the corresponding measurement and Z-score at the visit were set to missing. WLZ could not be obtained for infants with a length of less than 45.0 cm at any visit due to limitations in the WHO standards and were set to missing for that visit (1).

Arm 1 maternal participants received the study supplement starting at least 3 months prior to conception and continued through delivery; Arm 2 started the study supplement at the end of the first trimester and continued through delivery; Arm 3 (Control) did not receive study supplement. The number of infants considered for the combined site analyses according to treatment arm were 755, 808, and 774 for Arms 1, 2, and 3, respectively.

**Birth length is the strongest predictor of linear growth status and stunting in the first 2 years of life after a preconception maternal nutrition intervention: the children of the Women First trial (Krebs, et al)**

**Online Supplementary Material**

**Supplemental Table 3.** Adjusted linear growth outcomes comparisons from birth to 24 months by treatment arm and visit time point [1, 2]

| Visit | Arm Difference | Democratic Republic of Congo Length[3] | | Democratic Republic of Congo Length-for-age Z-score (LAZ)[4] | | All Sites LAZ <-2[5] | |
|---|---|---|---|---|---|---|---|
| | | Adj. Mean Diff. (95% CI) | *P* value | Adj. Mean Diff. (95% CI) | *P* value | Adj. Relative Risk (95% CI) | *P* value |
| | Arm 1 vs.2 | 0.32 (-0.09, 0.73) | 0.129 | 0.18 (-0.03, 0.39) | 0.087 | 1.08 (0.86, 1.35) | 0.526 |
| <7 days | Arm 1 vs.3 | 0.69 (0.25, 1.13) | 0.0022 | 0.37 (0.14, 0.60) | 0.0014 | 0.75 (0.61, 0.92) | 0.0057 |
| | Arm 2 vs.3 | 0.37 (-0.02, 0.76) | 0.062 | 0.19 (-0.02, 0.39) | 0.072 | 0.69 (0.56, 0.85) | 0.0006 |
| | Arm 1 vs.2 | 0.28 (-0.15, 0.71) | 0.198 | 0.15 (-0.06, 0.36) | 0.172 | 1.15 (0.94, 1.40) | 0.181 |
| 1 month | Arm 1 vs.3 | 0.90 (0.43, 1.37) | 0.0002 | 0.49 (0.26, 0.73) | <0.0001 | 0.87 (0.72, 1.04) | 0.127 |
| | Arm 2 vs.3 | 0.62 (0.21, 1.03) | 0.0032 | 0.35 (0.14, 0.55) | 0.0009 | 0.76 (0.63, 0.91) | 0.0038 |
| | Arm 1 vs.2 | 0.72 (0.29, 1.14) | 0.001 | 0.27 (0.07, 0.48) | 0.0097 | 0.94 (0.77, 1.15) | 0.540 |
| 3 months | Arm 1 vs.3 | 1.22 (0.78, 1.67) | <0.0001 | 0.56 (0.35, 0.77) | <0.0001 | 0.77 (0.63, 0.93) | 0.0074 |
| | Arm 2 vs.3 | 0.51 (0.08, 0.94) | 0.0197 | 0.29 (0.09, 0.50) | 0.0053 | 0.82 (0.68, 0.99) | 0.0343 |
| | Arm 1 vs.2 | 0.41 (-0.01, 0.83) | 0.057 | 0.16 (-0.03, 0.35) | 0.097 | 0.91 (0.77, 1.08) | 0.282 |
| 6 months | Arm 1 vs.3 | 0.99 (0.54, 1.45) | <0.0001 | 0.45 (0.24, 0.65) | <0.0001 | 0.76 (0.64, 0.89) | 0.0008 |
| | Arm 2 vs.3 | 0.59 (0.15, 1.03) | 0.0087 | 0.28 (0.08, 0.48) | 0.0056 | 0.83 (0.71, 0.97) | 0.0182 |
| | Arm 1 vs.2 | 0.46 (-0.05, 0.97) | 0.075 | 0.16 (-0.04, 0.37) | 0.124 | 0.93 (0.85, 1.03) | 0.179 |
| 12 months | Arm 1 vs.3 | 0.88 (0.35, 1.40) | 0.0012 | 0.36 (0.15, 0.57) | 0.0009 | 0.92 (0.83, 1.01) | 0.081 |
| | Arm 2 vs.3 | 0.41 (-0.10, 0.92) | 0.113 | 0.20 (-0.01, 0.40) | 0.061 | 0.98 (0.89, 1.08) | 0.673 |
| | Arm 1 vs.2 | 0.87 (0.25, 1.49) | 0.0059 | 0.30 (0.08, 0.52) | 0.0085 | 0.95 (0.88, 1.02) | 0.167 |
| 18 months | Arm 1 vs.3 | 1.04 (0.39, 1.69) | 0.0017 | 0.38 (0.15, 0.61) | 0.0012 | 0.94 (0.87, 1.01) | 0.101 |
| | Arm 2 vs.3 | 0.17 (-0.46, 0.79) | 0.610 | 0.08 (-0.14, 0.31) | 0.467 | 0.99 (0.92, 1.06) | 0.767 |
| 24 months | Arm 1 vs.2 | 0.81 (0.13, 1.50) | 0.0205 | 0.27 (0.05, 0.49) | 0.0172 | 0.96 (0.89, 1.02) | 0.181 |

| | | | | | | |
|---|---|---|---|---|---|---|
| Arm 1 vs.3 | 0.76 (0.07, 1.45) | 0.0298 | 0.23 (0.01, 0.46) | 0.037 | 0.95 (0.89, 1.02) | 0.186 |
| Arm 2 vs.3 | -0.05 (-0.72, 0.62) | 0.885 | -0.03 (-0.25, 0.18) | 0.760 | 1.00 (0.94, 1.06) | 0.980 |

[1] After excluding extreme invalid measurements as determined by expert manual review and accounting for biologically implausible Z-scores based on WHO standards [b], the 24-month longitudinal analysis subset includes all live born infants with birth length measurements measured by 7 days (168 hours) of age on portable length boards, and consented to the offspring follow-up study.

[2] All Z-scores were calculated using the expanded tables of the Child Growth Standards published by the WHO that provide Z-scores by sex and age in days at time of measurement. Weight-for-length Z-scores (WLZ) were calculated using the expanded tables of the Child Growth Standards published by the WHO that provide Z-scores by sex and tabulated lengths from 45.0 cm to 110.0 cm. All WHO standards are based on term infants. LAZ, WAZ, and WLZ were within the biologically plausible range according to WHO standards ($-6 \leq LAZ \leq 6$, $-6 \leq WAZ \leq 6$, $-5 \leq WLZ \leq 5$). If an infant was found to have a biologically implausible LAZ or WAZ according to WHO standards at a visit, all growth outcomes at the visit were set to missing. If an infant was found to have a biologically implausible WLZ according to WHO standards at a visit, only the corresponding measurement and Z-score at the visit was set to missing (1).

[3] Using longitudinal generalized linear models with generalized estimating equations (GEE), assuming a normal distribution and the identity link, and Auto-regressive order 1 covariance structure. Adjusted for country, cluster, visit, maternal education, stunting, parity, social-economic status (SES), infant sex, and arm by visit time interaction arm by visit time interaction *P* value was 0.011.

[4] Using longitudinal generalized linear models with GEE, assuming a normal distribution and the identity link, and Auto-regressive order 1 covariance structure. Adjusted for country, cluster nested within country, visit, maternal education, stunting, parity, SES, infant sex, and arm by visit time interaction. Arm by visit time interaction *P* value was 0.005.

[5] Using longitudinal generalized linear models with GEE, assuming a binary distribution and the log link, and Auto-regressive order 1 covariance structure. Adjusted for country, visit, maternal education, parity, SES, infant sex, and arm by visit time interaction. Arm by visit time interaction *P* value was 0.014.

Arm 1 maternal participants received the study supplement starting at least 3 months prior to conception and continued through delivery; Arm 2 started the study supplement at the end of the first trimester and continued through delivery; Arm 3 (Control) did not receive study supplement.

**Birth length is the strongest predictor of linear growth status and stunting in the first 2 years of life after a preconception maternal nutrition intervention: the children of the Women First trial (Krebs, et al)**

**Online Supplementary Material**

**Supplemental Table 4.** Spearman correlations for subjects included in the model for predictors of linear growth status at 24 months (WHO, n=2221)

| | SES | Maternal Educ | Maternal Stunting | Parity | Sex | Maternal BMI Cat | Maternal Ht Cat | Maternal Age Cat | Paternal Ht Cat | Paternal BMI Cat | Birth LAZ<-1 | Birth LAZ<-2 | Birth WAZ<-2 | Birth WLZ<-2 | LBW | Birth Stunting Group |
|---|---|---|---|---|---|---|---|---|---|---|---|---|---|---|---|---|
| **SES** | 1.000 | 0.362 | -0.196 | 0.018 | -0.030 | 0.202 | -0.157 | -0.004 | -0.056 | 0.214 | -0.079 | -0.028 | 0.021 | 0.034 | -0.009 | -0.078 |
| **Maternal Education** | 0.362 | 1.000 | -0.098 | -0.023 | 0.000 | 0.127 | -0.049 | -0.098 | -0.006 | 0.159 | -0.041 | -0.037 | -0.015 | -0.061 | -0.025 | -0.045 |
| **Maternal Stunting** | -0.196 | -0.098 | 1.000 | -0.104 | -0.001 | -0.285 | 0.922 | -0.021 | 0.252 | -0.148 | -0.120 | -0.021 | 0.003 | 0.068 | 0.020 | -0.114 |
| **Parity** | 0.018 | -0.023 | -0.104 | 1.000 | -0.007 | 0.121 | -0.108 | 0.361 | -0.072 | 0.053 | -0.088 | -0.029 | -0.183 | -0.107 | -0.187 | -0.087 |
| **Sex** | -0.030 | 0.000 | -0.001 | -0.007 | 1.000 | -0.033 | 0.002 | -0.001 | 0.003 | 0.018 | -0.046 | -0.069 | -0.022 | -0.008 | 0.034 | -0.056 |
| **Maternal BMI Cat** | 0.202 | 0.127 | -0.285 | 0.121 | -0.033 | 1.000 | -0.259 | 0.077 | -0.196 | 0.281 | -0.020 | 0.000 | -0.094 | -0.093 | -0.125 | -0.018 |
| **Maternal Ht Cat** | -0.157 | -0.049 | 0.922 | -0.108 | 0.002 | -0.259 | 1.000 | -0.020 | 0.224 | -0.118 | -0.141 | -0.037 | -0.001 | 0.049 | 0.011 | -0.137 |
| **Maternal Age Cat** | -0.004 | -0.098 | -0.021 | 0.361 | -0.001 | 0.077 | -0.020 | 1.000 | -0.034 | 0.086 | -0.021 | -0.006 | -0.069 | -0.025 | -0.070 | -0.020 |
| **Paternal Ht Cat** | -0.056 | -0.006 | 0.252 | -0.072 | 0.003 | -0.196 | 0.224 | -0.034 | 1.000 | -0.157 | -0.083 | -0.036 | 0.011 | 0.041 | 0.029 | -0.083 |
| **Paternal BMI Cat** | 0.214 | 0.159 | -0.148 | 0.053 | 0.018 | 0.281 | -0.118 | 0.086 | -0.157 | 1.000 | -0.047 | -0.018 | -0.076 | -0.059 | -0.082 | -0.046 |
| **Birth LAZ <-1** | -0.079 | -0.041 | -0.120 | -0.088 | -0.046 | -0.020 | -0.141 | -0.021 | -0.083 | -0.047 | 1.000 | 0.323 | 0.331 | -0.016 | 0.359 | 0.980 |
| **Birth LAZ <-2** | -0.028 | -0.037 | -0.021 | -0.029 | -0.069 | 0.000 | -0.037 | -0.006 | -0.036 | -0.018 | 0.323 | 1.000 | 0.316 | 0.049 | 0.313 | 0.503 |
| **Birth WAZ <-2** | 0.021 | -0.015 | 0.003 | -0.183 | -0.022 | -0.094 | -0.001 | -0.069 | 0.011 | -0.076 | 0.331 | 0.316 | 1.000 | 0.397 | 0.796 | 0.369 |
| **Birth WLZ <-2** | 0.034 | -0.061 | 0.068 | -0.107 | -0.008 | -0.093 | 0.049 | -0.025 | 0.041 | -0.059 | -0.016 | 0.049 | 0.397 | 1.000 | 0.397 | -0.004 |
| **LBW** | -0.009 | -0.025 | 0.020 | -0.187 | 0.034 | -0.125 | 0.011 | -0.070 | 0.029 | -0.082 | 0.359 | 0.313 | 0.796 | 0.397 | 1.000 | 0.393 |
| **Birth Stunting Group** | -0.078 | -0.045 | -0.114 | -0.087 | -0.056 | -0.018 | -0.137 | -0.020 | -0.083 | -0.046 | 0.980 | 0.503 | 0.369 | -0.004 | 0.393 | 1.000 |

BMI, body mass index; Cat, category; Educ, education; Ht, height; LAZ, length-for-age Z-score; Mat, maternal; SES, socio-economic status; SGA, small-for-gestational-age; WAZ, weight-for-length Z-score; WLZ, weight-for-length Z-score.

**Birth length is the strongest predictor of linear growth status and stunting in the first 2 years of life after a preconception maternal nutrition intervention: the children of the Women First trial (Krebs, et al)**

**Online Supplementary Material**

**Supplemental Table 5.** Spearman Correlations for subjects included in in the model for predictors of linear growth status at 24 months (Intergrowth-21st, n= 1329)

| | SES | Maternal Education | Maternal Stunting | Parity | Sex | Maternal BMI Cat | Maternal Ht Cat | Maternal Age Cat | Paternal Ht Cat | Paternal BMI Cat | Birth LAZ<-2 | Birth LAZ<-1 | SGA | Preterm | Birth Stunting Group |
|---|---|---|---|---|---|---|---|---|---|---|---|---|---|---|---|
| **SES** | 1.000 | 0.378 | -0.089 | 0.052 | -0.038 | 0.169 | -0.057 | -0.024 | -0.022 | 0.172 | -0.068 | -0.004 | 0.015 | -0.095 | -0.019 |
| **Maternal Education** | 0.378 | 1.000 | -0.051 | 0.002 | 0.001 | 0.118 | -0.017 | -0.074 | 0.015 | 0.171 | -0.055 | -0.020 | -0.082 | -0.142 | -0.030 |
| **Maternal Stunting** | -0.089 | -0.051 | 1.000 | -0.110 | -0.004 | -0.272 | 0.936 | -0.025 | 0.251 | -0.095 | -0.104 | -0.167 | 0.031 | 0.067 | -0.170 |
| **Parity** | 0.052 | 0.002 | -0.110 | 1.000 | -0.006 | 0.151 | -0.105 | 0.381 | -0.083 | 0.077 | -0.110 | -0.077 | 0.130 | -0.115 | -0.094 |
| **Sex** | -0.038 | 0.001 | -0.004 | -0.006 | 1.000 | -0.042 | -0.015 | 0.002 | -0.031 | -0.003 | 0.058 | 0.056 | -0.041 | -0.084 | 0.063 |
| **Maternal BMI Cat** | 0.169 | 0.118 | -0.272 | 0.151 | -0.042 | 1.000 | -0.246 | 0.103 | -0.180 | 0.282 | -0.102 | -0.062 | 0.182 | -0.053 | -0.079 |
| **Maternal Ht Cat** | -0.057 | -0.017 | 0.936 | -0.105 | -0.015 | -0.246 | 1.000 | -0.020 | 0.230 | -0.062 | -0.106 | -0.164 | 0.035 | 0.048 | -0.168 |
| **Maternal Age Cat** | -0.024 | -0.074 | -0.025 | 0.381 | 0.002 | 0.103 | -0.020 | 1.000 | -0.039 | 0.111 | -0.091 | -0.026 | 0.070 | -0.018 | -0.044 |
| **Paternal Ht Cat** | -0.022 | 0.015 | 0.251 | -0.083 | -0.031 | -0.180 | 0.230 | -0.039 | 1.000 | -0.131 | -0.069 | -0.110 | 0.046 | 0.004 | -0.112 |
| **Paternal BMI Cat** | 0.172 | 0.171 | -0.095 | 0.077 | -0.003 | 0.282 | -0.062 | 0.111 | -0.131 | 1.000 | -0.076 | -0.085 | 0.121 | -0.006 | -0.092 |
| **Birth LAZ <-2** | -0.068 | -0.055 | -0.104 | -0.110 | 0.058 | -0.102 | -0.106 | -0.091 | -0.069 | -0.076 | 1.000 | 0.451 | -0.405 | 0.027 | 0.634 |
| **Birth LAZ <-1** | -0.004 | -0.020 | -0.167 | -0.077 | 0.056 | -0.062 | -0.164 | -0.026 | -0.110 | -0.085 | 0.451 | 1.000 | -0.508 | -0.011 | 0.976 |
| **SGA** | 0.015 | -0.082 | 0.031 | 0.130 | -0.041 | 0.182 | 0.035 | 0.070 | 0.046 | 0.121 | -0.405 | -0.508 | 1.000 | 0.078 | -0.539 |
| **Preterm** | -0.095 | -0.142 | 0.067 | -0.115 | -0.084 | -0.053 | 0.048 | -0.018 | 0.004 | -0.006 | 0.027 | -0.011 | 0.078 | 1.000 | -0.003 |
| **Birth Stunting Group** | -0.019 | -0.030 | -0.170 | -0.094 | 0.063 | -0.079 | -0.168 | -0.044 | -0.112 | -0.092 | 0.634 | 0.976 | -0.539 | -0.003 | 1.000 |

BMI, body mass index; Cat, category; Educ, education; LAZ, length-for-age Z-score; Mat, maternal; SES, socio-economic status; SGA, small-for-gestational age; WAZ, weight-for-length Z-score; WLZ, weight-for-length Z-score.

**Birth length is the strongest predictor of linear growth status and stunting in the first 2 years of life after a preconception maternal nutrition intervention: the children of the Women First trial (Krebs, et al)**

**Online Supplementary Material**

**Supplemental Figure 1.** Consort diagram: All sites screening, consent, randomization, and longitudinal analysis subset by treatment arm

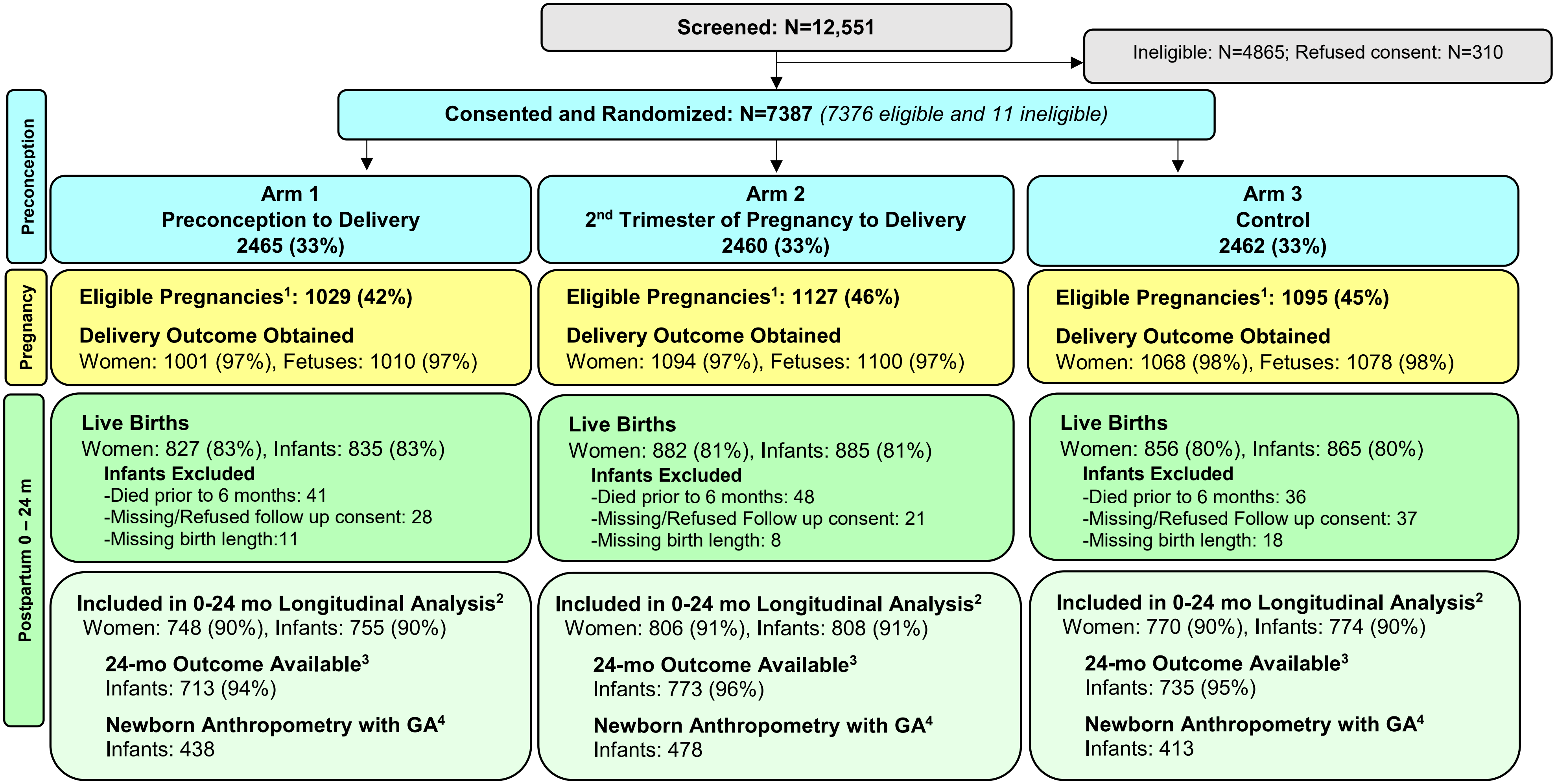

[1] Excludes women who became pregnant less than 3 months into the study. The women who had eligible pregnancies may have had delivery data obtained or they may have exited the study prior to delivery.

[2] After excluding extreme invalid measurements as determined by expert manual review and accounting for biologically implausible Z-scores based on WHO standards (1), the 24-month longitudinal analysis subset includes all live born infants with birth length measurements measured by 7 days (168 hours) of age on portable length boards, and consented to the offspring follow-up study.

[3] At least one of the following anthropometric outcomes must have been available after excluding extreme invalid measurements as determined by expert manual review and accounting for biologically implausible Z-scores based on WHO standards:(1) length, weight, head-circumference, weight-for-length measurements.

[4] Gestational age (GA) at birth is defined as the GA determined by ultrasound based on the ultrasound plus time until birth if the ultrasound was done between 6 weeks + 0 days to 13 weeks + 6 days, and the GA at birth is between 24 weeks + 0 days and 42 weeks + 6 days. If the ultrasound was not conducted during the GA interval previously mentioned, then the GA at birth is missing. Please note, ultrasounds were not performed in the Democratic Republic of Congo.

**Birth length is the strongest predictor of linear growth status and stunting in the first 2 years of life after a preconception maternal nutrition intervention: the children of the Women First trial (Krebs, et al)**

**Online Supplementary**

**Supplemental Figure 2.** Proportion of 24-month-old participants with length-for-age Z-score (LAZ) <-2 by research site and arm

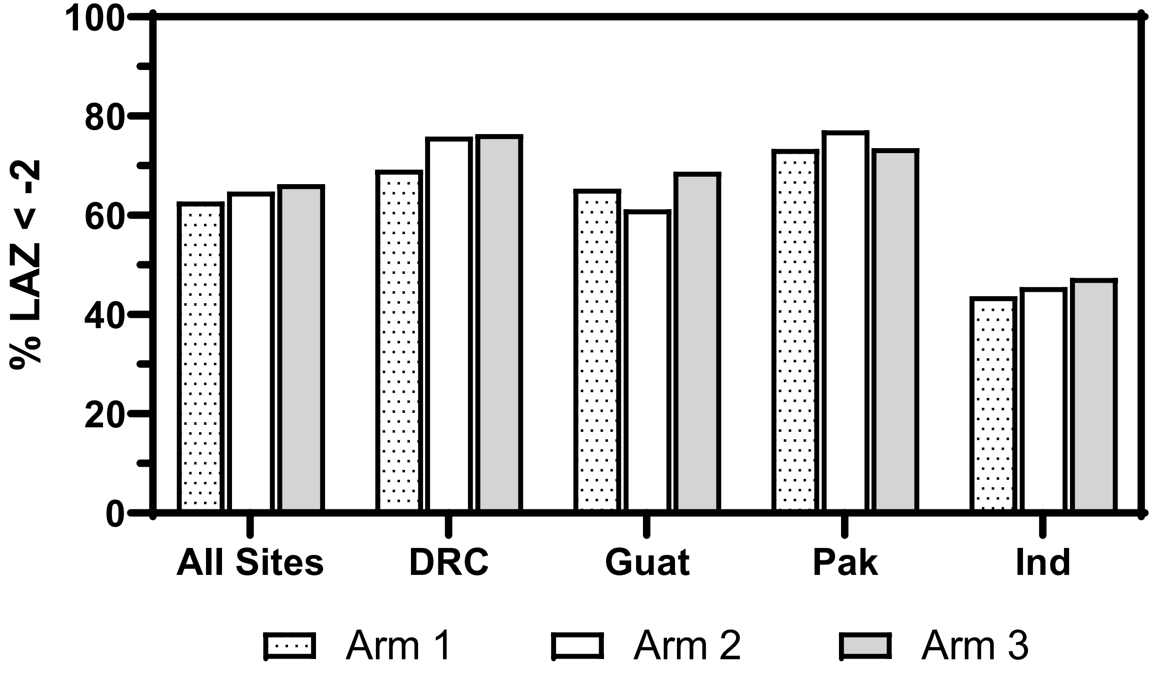


Data for all sites are available in Supplemental Table 1. Rates of stunting at 24 months by unadjusted LAZ did not differ by arm.

Arm 1 maternal participants received the study supplement starting at least 3 months prior to conception and continued through delivery; Arm 2 started the study supplement at the end of the first trimester and continued through delivery; Arm 3 (Control) did not receive study supplement. The number of infants considered for the combined site analysis according to treatment arm were 713, 773, and 735 for Arms 1, 2, and 3, respectively.

DRC, Democratic Republic of Congo; Guat, Guatemala; Pak, Pakistan; Ind, India

1. World Health Organization. The WHO Child Growth Standards: Length/height-for-age, weight-for-age, weight-for-height and body mass index-for-age. Methods and development. Geneva: WHO, 2006.
